# Variation in Melatonin Contents and Genetic Dissection of Melatonin Biosynthesis in Sesame

**DOI:** 10.3390/plants11152005

**Published:** 2022-07-31

**Authors:** Xiao Wang, Jun You, Aili Liu, Xin Qi, Donghua Li, Ya Zhao, Yanxin Zhang, Liangxiao Zhang, Xiurong Zhang, Peiwu Li

**Affiliations:** 1 Oil Crops Research Institute, Chinese Academy of Agricultural Sciences, Wuhan 430062, China; wangxiao0613@163.com (X.W.); liuailihappy@126.com (A.L.); qixin@caas.cn (X.Q.); lidonghua@caas.cn (D.L.); zhaoya1109@163.com (Y.Z.); zhangyanxin@caas.cn (Y.Z.); 2 Key Laboratory of Biology and Genetic Improvement of Oil Crops, Ministry of Agriculture and Rural Affairs, Wuhan 430062, China; 3 Quality Inspection and Test Center for Oilseed Products, Ministry of Agriculture and Rural Affairs, Wuhan 430062, China; 4 Hubei Hongshan Laboratory, Wuhan 430070, China

**Keywords:** melatonin, sesame, LC-MS/MS, GWAS

## Abstract

In recent years, people have become increasingly interested in bioactive molecules in plants that are beneficial to human health, and melatonin (N-acetyl-5-methoxytryptamine) has attracted research attention due to its excellent performance. In this study, the content of melatonin in oilseeds was investigated. From the results, it was found that sesame is an important natural food source of melatonin intake. Furthermore, the variation in melatonin content was explored in a natural sesame population, and its contents varied from 0.04 to 298.62 ng g^−1^. Through a genome-wide association study (GWAS), a candidate gene *SiWRKY67* was screened that regulates melatonin content in sesame. The sesame hairy root transformation system was developed and used to verify this gene, and it was found that the overexpression of *SiWRKY67* could positively promote the melatonin content in the hairy roots. Our results provide not only a foundation for understanding the genetic structure of melatonin content in sesame seeds but also a reference for the marker-assisted breeding of sesame varieties with high melatonin content.

## 1. Introduction

As a well-known anti-oxidative, anti-inflammatory and anti-viral molecule, melatonin (N-acetyl-5-methoxytryptamine) has drawn research interest [1,2]. Melatonin is widely believed to be a beneficial bioactive molecule with health-promoting effects. Melatonin possesses many physiological functions, including circadian rhythm regulation to promote sleep [3], and provides an antioxidant effect by scavenging free radicals and reducing oxidative damage [4], as a potential treatment for osteoporosis [5], neurodegenerative diseases [6], diabetes [7] and cancer. Melatonin is an evolutionary conservative molecule that has existed for billions of years. With the exception of the pineal gland in mammals and humans, melatonin is found ubiquitously in plants, where it has various physiological effects including, but not limited to, regulating photoperiod, participating in growth regulation, removing active oxygen and enhancing anti-oxidation enzyme activity [8]. Compared with melatonin extracted from the pineal gland or produced through chemosynthesis, plant-derived melatonin is more easily accepted by consumers, who feel comfortable consuming it. Moreover, it has the advantage of a synergistic effect with other nutrients ingested at the same time, and it does not introduce pollutants because it is not chemically synthesized. Melatonin has been reported to be found in many plants and their products, including the root, stem, leaf, fruit, seed and other tissues of higher plants, including vegetables and fruits, herbal medicines, maize, wheat and oats [9]. Of the high-melatonin foods, such as grape, cherry, ginger, tomato and sesame [10,11,12], sesame contains the highest content of melatonin and can therefore be an ideal source of plant-derived melatonin intake.

Sesame (*Sesamum indicum* L.) is an important oil crop that has been cultivated for more than 2000 years [13]. Due to its unique fragrance and various nutrients, sesame and its products are very popular in China, Korea, Japan and other Asian countries. The world’s annual sesame planting area is about 12.8 million hm^2^, and the output is about 6.5 million tons [14]. In traditional Chinese and Indian medicine, sesame has been used in the treatment of central nervous system disorders and insomnia. Pre-experiment results indicated that sesame is an important natural source of melatonin in food. Improvement of the melatonin content in sesame could improve melatonin intake in human beings; however, the biosynthesis regulation mechanism of melatonin in sesame is still unclear [15]; therefore, it is necessary to determine the genetic regulatory mechanisms of melatonin formation in sesame in order to cultivate new high-melatonin sesame varieties.

Genome-wide association study (GWAS) involves the positive genetic analysis of complex traits, utilizing correlation analysis of alternative genetic variation and phenotypic variation on all individual genome levels in a group to identify significant chains and analyze the genetic effects of allelicity on the phenotype [16,17,18]. To date, GWAS has been successfully applied to the analysis of the genetic bases of metabolome variation in soybean [19] and the correlation analysis of metabolites in tomatoes [20]. At present, more than 20,000 sesame germplasm resources have been collected and preserved all over the world, of which 8115 basic sesame collections have been preserved in the China National Gene Bank [21]. In our previous study, 705 diverse sesame varieties were sequenced to construct a haplotype map of the sesame genome and de novo assemble two representative varieties [22]. Moreover, 56 agronomic traits, including oil content and fatty acid composition, were investigated by GWAS to discover the candidate causative genes.

In this study, the melatonin contents of 269 traditional landraces and 80 modern cultivars from China, as well as 101 accessions collected from 26 other countries, were determined by liquid chromatography-tandem mass spectrometry (LC-MS/MS) to reveal the melatonin content distribution in sesame. By exploiting the natural variation in these 450 sesame genomes and performing a large-scale GWAS on melatonin, key genomic loci were systemically identified for the first time. These genetic discoveries may potentially be used to further breed high-melatonin sesame and also provide important guidance for improving the melatonin content in other crops.

## 2. Results

### 2.1. Quantitative Analysis of Melatonin

The improved QuEChERS (Quick, Easy, Cheap, Effective, Rugged and Safe) method has typically been used to extract targets from samples. Most studies have used methanol or acetonitrile as the extraction solvent. In order to select a suitable extraction solvent, the extraction efficiency of methanol and acetonitrile was compared. As shown in Figure S1, methanol was selected as the extraction solvent in the dark, as melatonin is prone to photodegradation. Next, the liquid ratio was optimized. Then, C18 and primary secondary amine (PSA) dispersant were used to remove the interference of pigments and other matrix interferences. The average recovery of the sample purified by C18 was better than by PSA; therefore, C18 was selected as the purification dispersant for the experiment. The amount of C18 dispersant was optimized, and the experimental results are shown in Figure S1. Ultimately, 10 mL of methanol was the optimal extraction reagent, and 600 mg of C18 was the optimal purifier.

The molecular weight of melatonin is 232. The parent ion [M+H]+ after protonation was observed at m/z 233 in the positive ion mode. NH4C2O would be lost during subsequent collisions, forming a major fragment m/z 174, selected as a qualitative ion in Figure S2. The calibration curve was constructed by using the standard solution series (triplicates) of melatonin. The limit of detection (LOD) and limit of quantification (LOQ) were evaluated based on a signal-to-noise ratio (S/N) of 3 or 10, respectively. The LOD and LOQ were 0.03 µg/L and 0.09 µg/L, respectively. Excellent linearity was obtained for melatonin within a range of 1–1000 μg/L. A typical regression equation was obtained with a correlation coefficient of 0.999. The validation results showed that the recovery rates of standard addition ranged from 92.14% to 97.00%. The foregoing findings suggested that the proposed approach might be used to detect melatonin in oilseeds. The optimized and improved QuEChERS was used to extract and purify melatonin from oilseeds, and LC-MS/MS in MRM mode was employed to determine the melatonin.

### 2.2. Comparison of Melatonin Contents in Different Oilseeds and Common Plant-Derived Foods

To investigate the melatonin content of oilseeds, sesame, rapeseed, soybean, walnut, camellia seed, maize, peanut, wheat and rice were collected and analyzed using the analytical method developed in this study. A total of 81 edible oilseed samples were analyzed in triplicate. The average value of each type of oilseed was calculated. Melatonin was detected only in sesame, maize, walnut and rice, and the average contents were 7.06 ng g^−1^, 1.52 ng g^−1^, 3.60 ng g^−1^ and 1.00 ng g^−1^, respectively. The contents of melatonin in different varieties of sesame seeds were also quite different, ranging from 0.41 ng g^−1^ to 25.18 ng g^−1^, which were significantly higher than those in the other oilseeds. Moreover, we summarized the melatonin content in plant-derived foods, including common fruits, vegetables, grains and oilseeds. The results indicated that most vegetables and fruits had low levels of melatonin, except for cherry (13.46 ng g^−1^). Compared with vegetables and fruits, some oilseeds contained a high content of melatonin, such as sesame seed, sunflower seed (29 ng g^−1^) and flaxseed (12 ng g^−1^) in Table 1. Because of the high contents of melatonin in sesame, it can be used as a dietary supplement for melatonin; however, to date, high-melatonin sesame is still scarce; therefore, it is necessary to reveal the biosynthesis and regulatory mechanism of melatonin in sesame for breeding new high-melatonin sesame varieties.

### 2.3. Distribution of Melatonin Content in Sesame Germplasm Resources

Differences in melatonin content are influenced not only by plant species, but also by cultivated varieties within the same plant species. In order to explore the differences in melatonin content among sesame genotypes and select high-melatonin sesame resources, we collected 450 and 392 sesame germplasm resources from 27 countries and planted them in Hubei (HB) and Shandong (SD), respectively.

**Figure 1:**
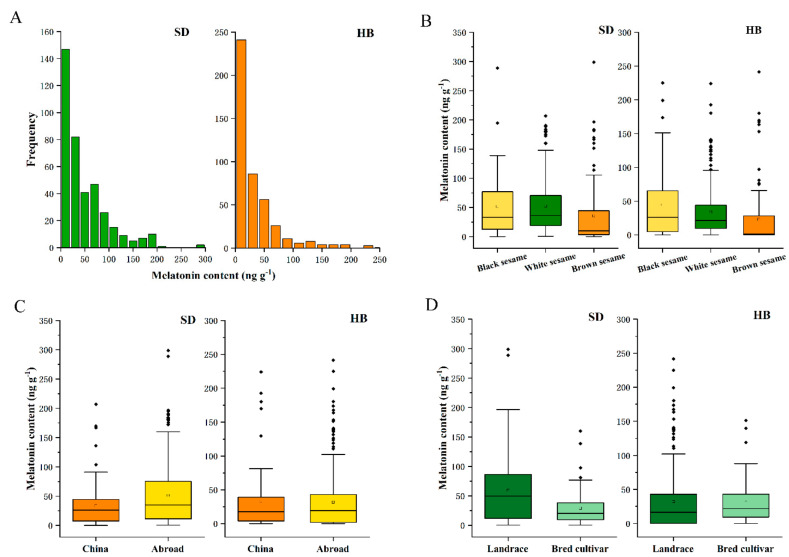
(**A**) Distributions of melatonin contents in sesames from SD and HB. (**B**) The melatonin content of different seed colors is shown in boxplot graph. (**C**) The boxplot graph of melatonin content from China and abroad. (**D**) The melatonin content of landrace and bred cultivar in boxplot graph.

The melatonin contents of 842 sesame seeds, including 450 sesame seeds in HB and 392 sesame seeds in SD were determined by LC-MS/MS. The content distribution is shown in Figure 1A. The average melatonin content in SD was 47.6 ng g^−1^, while the average of HB was 31.7 ng g^−1^. The contents of 49 accessions from Shandong were more than 100 ng g^−1^; however, only 30 of those from HB had more than 100 ng g^−1^. This difference may be caused by environmental factors, as the level of secondary metabolites is greatly affected by the environment [32]. The G118 genotype had the highest melatonin content, of 298.62 ng g^−1^, in SD, while the highest melatonin content of G118 in HB was 241.39 ng g^−1^. The melatonin content in the different varieties of sesame varied greatly. The melatonin content in plant-derived foods is largely determined by genetic characteristics [33]. Genetic traits are the basis of a species and are the determinants of the overall differences between plants, including melatonin content. Clearly, this feature emphasizes the importance of cultivating and harvesting multiple plants with the highest melatonin concentrations in order to obtain natural, safe and effective melatonin for human use.

Based on the color of sesame seeds, sesame samples were roughly divided into black, white and brown. The box plots of their content distribution are shown in Figure 1B, with the mean values of 51.66, 52.36 and 35.71 ng g^−1^ in SD. The mean values in HB were 44.38, 33.36 and 22.88 ng g^−1^. The melatonin content in brown sesame is lower than that in white and black sesame. Intermediates and final metabolites affect not only the quality of edible oils, but also the seed coat color of crops [34]. Melatonin, as a derivative of tryptophan, is a multi-regulatory factor that can coordinate all aspects of plant development [35] and may also be involved in the regulation of seed color. According to their origin, sesame samples were divided into Chinese sesame and foreign sesame, as shown in Figure 1C. In SD, melatonin levels in Chinese and foreign sesame samples were 33.69 ng g^−1^ and 51.26 ng g^−1^, respectively. In HB, melatonin levels in Chinese and foreign sesame samples were 33.32 ng g^−1^ and 31.64 ng g^−1^, respectively. Among the numerous samples collected from various sources, Chinese sesame was separated into landrace and bred cultivars, as indicated in Figure 1D. In SD, the average melatonin concentrations of local and bred types were 59.45 and 29.08 ng g^−1^, respectively, while in HB, they were 32.03 and 30.92 ng g^−1^. Landraces, however, have a higher melatonin concentration than bred cultivars. Melatonin is resistant to abiotic stress factors, and high-yield varieties with good resistance can be bred through a directional breeding process; however, melatonin produced through directional breeding processes showed a degradative trend.

### 2.4. GWAS Study for Melatonin in Sesame

**Figure 2:**
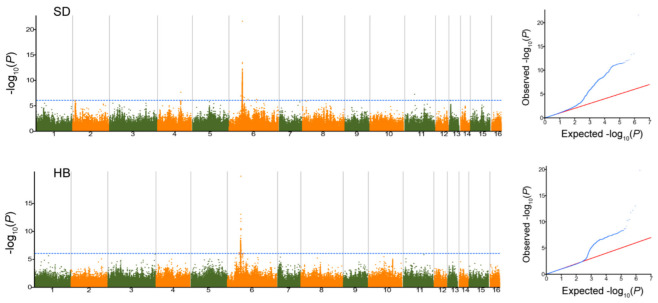
Manhattan and Q-Q plots of genome-wide association studies for melatonin content in two environments (SD and HB). The dashed blue line represents the significance threshold (−log10 *p* = 6.0).

To obtain insight into the genetic variants underlying the melatonin content in sesame, GWAS based on the EMMAX model was conducted using one million genome-wide SNPs [22]. The Manhattan and QQ (quantile-quantile) diagrams for the melatonin content in SD and HB are shown in Figure 2. A total of three significant associations located on LG2, LG4 and LG6 were identified for melatonin content in the two environments at *p* < 1 × 10^−6^ (Figure 2). The strongest association for melatonin content represented by SNP6_6839561 (*p* < 1.5 × 10^−20^) was detected on LG6 in both SD and HB and contributed 21.4–24.4% of the phenotypic variation in the entire population. The remaining significant associations were detected in SD and explained 11.7–15.3% of the total phenotypic variation.

### 2.5. Genes Underlying Melatonin Content in Sesame

According to the estimated linkage disequilibrium (LD) decay in the sesame genome [22], a total of 14 potential candidate genes related to sesame melatonin content were detected within 88 kb upstream and downstream of the peak SNP (SNP6_6839561) on LG6. Among these candidate genes, the expression of 10 genes was detected in nine seed development stages in sesame (Figure S3). Six genes (LOC105163919, LOC105163920, LOC105163921, LOC105163923, LOC105163926 and LOC105163927) were highly expressed (FPKM value > 10) in at least five seed development stages. Some transcription factors have been demonstrated as upstream regulators of melatonin biosynthesis in plants. For example, tomato heat-shock factor A1a (HsfA1a) conferred cadmium tolerance by activating the transcription of the COMT1 gene and inducing melatonin biosynthesis under cadmium stress [36]. In cassava, the WRKY transcription factor (MeWRKY79) and heat shock transcription factor (MeHsf20) were found to activate MeASMT2 and melatonin biosynthesis [37]. In another study, two RAV transcription factors (MeRAV1 and MeRAV2) were demonstrated to positively regulate the endogenous melatonin level and function in disease resistance against cassava bacterial blight [38]; therefore, one candidate gene encoding a WRKY transcription factor (LOC105163923), named SiWRKY67 according to a previous study [39], was chosen for functional verification.

Agrobacterium rhizogenes-induced hairy root systems are one of the most preferred and versatile systems for the functional characterization of genes [40]. Till now, Agrobacterium rhizogenes-induced hairy root systems are widely used for producing recombinant proteins and valuable bioactive compounds [41]. Hairy root transformation also provides an alternative tool for studying gene function in plants that are recalcitrant to transformation [42,43]. Recently, sesame hairy root culture has been reported to obtain recombinant fungal phytase and is used to analyze the secondary metabolic pathways [44]; therefore, in the present study, due to the difficulty in genetic transformation of sesame, Agrobacterium rhizogenes-mediated hairy root transformation was used to verify the function of SiWRKY67 for melatonin accumulation in sesame. Agrobacterium rhizogenes K599 carrying the SiWRKY67 gene was used to infect sesame cotyledon explant, and 28 hairy root lines were obtained. The lines with better growth statuses were selected for PCR and qRT-PCR analyses. Three independent transgenic hairy root lines with elevated SiWRKY67 expression were used for further study (Figure S4). The contents of melatonin in the SiWRKY67-OE hairy root lines and empty vector control (VC) were determined by UHPLC-MS/MS. The results showed that no melatonin was detected in VC, due to the concentration of melatonin being below the detection limit of the UHPLC-MS/MS system; however, the melatonin content in the SiWRKY67-OE hairy root lines ranged from 0.293 ng g^−1^ FW to 0.452 ng g^−1^ FW (Table 2), indicating that SiWRKY67 functions in melatonin accumulation in sesame. 

## 3. Discussions

In recent years, the bioactive components in natural plants have attracted more attention, especially antioxidants such as melatonin. Melatonin has various physiological functions and is very beneficial to human health. Although melatonin supplements are available on the market, most of them are chemically synthesized or extracted from the pineal gland, which always leads to the possibility of introducing pollutants [45]. Among them, 1,1′-ethylidenebis-(tryptophan), a contaminant introduced in the process of synthesizing melatonin, caused 27 deaths in the 1990s, and this compound is associated with the potential development of eosinophilia myalgia syndrome [46,47]. Compared with melatonin pills or capsules, melatonin in food is more easily accepted by consumers. In addition, phytomelatonin has the advantage of synergistic effects with other nutrients consumed at the same time. In this study, an LC-MS/MS method for the determination of melatonin was established and used to analyze nine common edible oilseeds. Compared with the content of melatonin in common plant-derived foods reported in the literature, sesame can be used as a good source of phytomelatonin supplement.

Internal and external factors may affect the melatonin content, mainly including genetic traits, phenological period of the cultivar, the photoperiod, planting environment and agrochemicals [9]. In order to explore the dominant germplasm of melatonin, the melatonin content of 842 sesame accessions collected from natural populations was determined. To the best of our knowledge, there has been no published work to date describing the melatonin content on such a large scale. The melatonin content varied greatly among varieties, with the highest content of 298.62 ng g^−1^ and the lowest content of 0.03 ng g^−1^. Among them, G180 with high melatonin content (288.82 ng g^−1^) was found through large-scale screening. Moreover, it is necessary to emphasize that G180 contains high content of sesame lignans (9.3 mg g^−1^), including sesamin (7.4 mg g^−1^) and sesamolin (1.9 mg g^−1^). The melatonin content was affected not only by genetic background, but also by the planting environment. The average melatonin contents in SD and HB were 47.6 and 31.7 ng g^−1^, respectively, indicating that Shandong Province might be suitable for the cultivation of sesame with high-content melatonin.

GWAS has become a popular and effective approach in plant genetic studies owing to the rapid advance of sequencing technology in recent years [48]. Sesame has an exceptionally large diversity within species, as well as rapid LD decay [22]. In this study, WRKY transcription factor (LOC105163923, named SiWRKY67) was identified as a candidate gene associated with melatonin content in sesame. The SiWRKY67 gene was verified by the hairy root transformation system, and it was found to have a positive regulatory effect on melatonin accumulation. Single-nucleotide polymorphisms of this WRKY transcription factor could be used as a molecular genetic marker of sesame with high melatonin content in molecular breeding. In the future, studying the regulation mechanism of the transcription factor to positively regulate melatonin accumulation may be important. Moreover, combining with other antioxidants such as sesamin and sesamolin, the development of double high (high melatonin and high lignan) sesame and its products may improve the anti-oxidative and anti-inflammatory effects of sesame.

## 4. Material and Methods

### 4.1. Chemicals and Standards

The organic solvents and reagents used were of LC-MS grade. Methanol, acetonitrile, formic acid and ethanol were purchased from Thermo Fisher Scientific (Shanghai, China). The standard of melatonin was of high purity grade (>98%) and purchased from Sigma-Aldrich (Merck KGaA, Darmstadt, Germany).

In total, 10 milligrams of melatonin standard were weighed and dissolved in 10 mL methanol. The 1000 μg/mL standard solution was diluted with methanol step by step to prepare a 10 μg/mL, 1 μg/mL, 500 μg/L, 100 μg/L, 10 μg/L and 1 μg/L standard solution series and the solutions were placed in the freezer at −20 °C for use.

### 4.2. Collection of Samples

Nine types of seeds, namely sesame, rapeseed, soybean, walnut, camellia seed, corn, peanut, wheat and rice, were collected from local markets and placed at 4 °C for later use.

All the sesame samples were extracted from 8115 sesame accessions preserved in the National Gene Bank of China, Oil Crops Research Institute, Chinese Academy of Agricultural Sciences. A total of 450 accessions were selected, namely, 269 traditional landraces and 80 modern cultivars, as well as 101 varieties from 26 other countries. Prior to the determination of melatonin, 450 accessions were preserved for at least four generations through self-pollination. For phenotyping, the 450 accessions were planted in Wuhan, Hubei Province (HB), China. Limited by the environmental adaptability of sesame, 392 out of 450 accessions were planted in Jinan, Shandong Province (SD), China. The sesame seeds were ground to a powder with a crusher and placed in a 4 °C refrigerator for later use. The list of 450 accessions sampled in the collection in Table S1.

### 4.3. Sample Extraction and Preparation

Approximately 1 g of sample was weighed and combined with 10 mL of methanol, vortexed and mixed at 4 °C overnight, and then shaken for 3.5 h under dark conditions. The sample was centrifuged at 4500 rpm for 5 min and added 600 mg C18 dispersant to supernatant and vibrated for 5 min to purify the sample. Then, the sample was centrifuged at 4500 rpm for 5 min and the supernatant was dried with liquid nitrogen, reconstituted in 0.5 mL methanol and passed through a 0.22 μm organic membrane for the subsequent analysis.

### 4.4. LC-MS/MS Analysis

Analysis of melatonin was performed on a UHPLC system equipped with a Shimadzu MS-8060 triple quadrupole mass spectrometer (Kyoto, Japan) with an electrospray ionization (ESI) source (Turbo Ionspray). The separation of MLT was achieved on a Thermofisher Hypersil GOLDTM C18 HPLC column (2.1 mm × 100 mm, 3 μm) with a flow rate of 0.25 mL/min at 40 °C. Solvent A was a methanol solution of 0.1% acetic acid and solvent B was an aqueous solution. The gradient elution program was as follows: 0–1 min, 95% A; 1–2.5 min, 95–80% A; 2.5–5 min, 80–65% A; 5–7.5 min, 65–10% A; 7.6–10 min, 95% A. An aliquot of 2 μL of sample was injected into the UHPLC-MS/MS system. In the positive ion mode, multiple reaction monitoring (MRM) was used to analyze the melatonin. The MRM transitions were m/z 233→174, 159 and 130 (see Figure S2), where m/z 174 was used as a quantitative ion. The UHPLC-MS/MS automated system was used to optimize the collision energy (CE). The best ESI source conditions were thermal resistance temperature 400 °C, DL temperature 250 °C, spray gas 3 L/min and dry gas 10 L/min. The identification of melatonin was accomplished by the comparison of retention time and MS2 with those of available authentic standard (Sigma-Aldrich, Merck KGaA, Darmstadt, Germany). The quantitative determination was conducted by the external standard method. The curve of the peak area of the quantified ion for known concentration was created with six calibration points. 

### 4.5. Population Genetics Analysis and GWAS Study

The association panel was re-sequenced as previously described [22]. A total of 1 M common single nucleotide polymorphisms (SNPs) with minor allele frequency >0.03 were used in this study. The GWAS was performed with the EMMAX software package [49] based on log2-transformed melatonin concentrations. The suggestive significance threshold was defined as *p* < 1 × 10−6 to determine significantly associated SNP markers, as in previous studies in sesame [50,51]. The phenotypic variance explained (PVE) of each peak SNP was determined using r2 values obtained from simple linear regressions.

### 4.6. Candidate Gene Mining

The potential candidate genes for melatonin content were retrieved in the 88 kb region corresponding to the average linkage disequilibrium window [22] around the significant peak SNP based on the sesame reference genome. In order to analyze candidate genes with higher gene expression levels in sesame seeds, the transcriptomic datasets of nine different seed developmental stages (5, 8, 11, 14, 17, 20, 23, 26 and 30 days after flowering) from our previous study were used [52]. The heatmaps were generated using TBtools software [53]. Candidate genes were retained based on the gene annotation and their expression levels in sesame seeds.

### 4.7. Plasmid Construction and Hairy Root Transformation

The full-length cDNA sequence of SiWRKY67 (LOC105163923) was obtained from NCBI. The complete open reading frame cDNA of SiWRKY67 was isolated from the seeds of the high-melatonin sesame genotype G118 by quantitative real-time (qRT)-PCR using the gene-specific primers SiWRKY67FL-F (AGCTTTCGCGAGCTCGGTACCATGTCTTCCGCC TCTTTCAC TAGT) and SiWRKY67FL-R (CAGGTCGACTCTAGAGGATCCTCAGCGG AGCAAAGACTCGA). The PCR product was then cloned into the vector pCAMBIA1301S under the control of a 2 × CaMV 35S promoter by homologous recombination. The pCAMBIA1301S-SiWRKY67 construct was confirmed by sequencing and transferred into Agrobacterium tumefaciens strain K599.

Because of the high hairy root induction rate and rapid hairy root growth during transformation, sesame genotype G98 from China was used for hairy root induction and transformation [54] with some modifications. Briefly, wounded cotyledons dissected from sterile sesame seedlings were infected with engineered K599 strains containing the pCAMBIA1301S-SiWRKY67 or empty vector. Infected explants were cultured on half-strength Murashige and Skoog (MS) solid medium supplemented with 100 mg/mL kanamycin and 200 mg/L timentin. Subsequently, the hairy roots were cut and moved to fresh half-strength MS solid medium every 2 weeks. The expression of SiWRKY67 in transgenic hairy root lines was confirmed by qRT-PCR using the gene-specific primers SiWRKY67QF (CCATCGCAACAACAATACTG) and SiWRKY67QR (CCATCATCGG ACCTTCTG), as previously described [55].

## 5. Conclusions

In this study, an improved LC-MS/MS method was used to determine melatonin in 842 sesame accessions in two environments. The specific sesame germplasm G118 with high melatonin content was screened out, which could be used as a reserve resource for breeding sesame with high melatonin content to directly develop a healthy food. To further improve the melatonin content in sesame, GWAS analysis of the melatonin content in a natural population of sesame was conducted to find a candidate gene SiWRKY67 that regulates melatonin content. The sesame hairy root transformation system was developed and used to verify it. The validation results indicated that the overexpression of SiWRKY67 could positively promote the melatonin content in the hairy roots; therefore, the genetic basis of melatonin content and the gene regulation of melatonin synthesis identified in sesame have important significance for cultivating new sesame varieties with high melatonin and enhancing the nutritional quality of sesame. Moreover, this study also provided a clue to investigating the genetic mechanism of indole derivatives related to melatonin.

## Figures and Tables

**Table 1:** The content of melatonin in plant-derived foods.

Common Name	Scientific Name	Melatonin	Region	Method	Ref.
Sesame	*Sesamum indicum* L.	0.41-25.18 ng g^−1^	China	LC-MS/MS	This study
Rapeseed	*Brassica campestris* L.	NA	China	LC-MS/MS	This study
Soybean	*Glycine max* (Linn.) Merr.	NA	China	LC-MS/MS	This study
Walnut	*Juglans regia* L.	3.60 ng g^−1^	China	LC-MS/MS	This study
Camellia seed	*Theaceae Camellia* L.	NA	China	LC-MS/MS	This study
Corn	*Zea mays* L.	1.52 ng g^−1^	China	LC-MS/MS	This study
Wheat	*Triticum aestivum* L.	NA	China	LC-MS/MS	This study
Rice	*Oryza sativa*	1.00 ng g^−1^	China	LC-MS/MS	This study
Sunflower seed	*Helianthus annuus* L.	29 ng g^−1^	America	HPLC-ECD	[23]
Flaxseed	*Linum usitatissimum* L.	12 ng g^−1^	America	HPLC-ECD	[24]
Poppy seed	*Popaver somniferum* L.	6 ng g^−1^	America	HPLC-ECD	[24]
Barley	*Hordeum vulgare* L.	0.87 ng g^−1^	Egypt	GC-MS	[25]
Corn	*Zea mays* L.	1.88 ng g^−1^	Egypt	GC-MS	[25]
Extra virgin olive oil	-	0.071–0.119 ng mL;^−1^	Spain	ELISA	[26]
Banana	*Musa sapientum* L.	0.47 ng g^−1^	Germany	RIA and GC-MS	[25]
Apple	*Malus domestica*	0.05 ng g^−1^	Japan	Radioimmunoassay	[27]
Strawberry	*Fragaria × ananassa* Duch	0.14 ng g^−1^	Egypt	GC-MS	[28]
Barley	*Hordeum vulgare* L.	0.87 ng g^−1^	Egypt	GC-MS	[25]
Orange	*Citrus reticulata Blanco*	0.15 ng g^−1^	Thailand	HPLC-FD & ELISA	[29]
Pineapple	*Ananus comosus* M.	0.28 ng g^−1^	Egypt	GC-MS	[25]
Montmorency tart cherry	*Prunus cerasus* L.	13.46 ng g^−1^	Germany	HPLC-ECD	[24]
Barbera grape (skin)	*Vitis vinifera* L.	0.63 ng g^−1^	Italy	HPLC-FD and ELISA	[30]
Mango	*Mangifera indica* L.	0.70 ng g^−1^	Thailand	HPLC-FD & ELISA	[29]
Papaya	*Carica papyya* L.	0.24 ng g^−1^	Thailand	HPLC-FD & ELISA	[29]
Cucumber	*Cucumis sativus* L.	0.02 ng g^−1^	Japan	Radioimmunoassay	[27]
Asparagus	*Asparagus officinalis* L.	0.01 ng g^−1^	Japan	Radioimmunoassay	[27]
Welsh onion	*Allium fistulosum* L.	0.09 ng g^−1^	Japan	Radioimmunoassay	[27]
Tomato	*Solanum lycopersicum* L.	0.30 ng g^−1^	Egypt	GC-MS	[25]
Ginger	*Zingiber officinale* R.	1.42 ng g^−1^	Egypt	GC-MS	[25]
Red wine	—	0.05–0.62 ng mL^−1^	Italy	UPLC-HRMS	[31]
White wine	—	0.18 ng mL^−1^	Italy	UPLC-HRMS	[31]

**Table 2:** Content of melatonin in *SiWRKY67*-OE hairy root lines.

Samples	Content of Melatonin (ngg^−1^ FW)	Range of Values (ng g^−1^ FW)
VC	ND	-
*SiWRKY67*-OE#1	0.329 ± 0.032	0.293–0.352
*SiWRKY67*-OE#2	0.406 ± 0.042	0.369–0.452
*SiWRKY67*-OE#3	0.350 ± 0.024	0.325–0.373

The content of melatonin was analyzed by LC-MS/MS and the data obtained from three replicates are presented as mean ± SD. VC: empty vector control. FW: fresh weight. ND: not detected.

## Data Availability

Not applicable.

## Supplementary Materials

The following supporting information can be downloaded at: https://www.mdpi.com/article/10.3390/plants11152005/s1, Figure S1. Optimization of sample pretreatment conditions (A) the extraction efficiency of Methanol (MeoH) and Acetonitrile (ACN)); (B) effect of extraction solvent MeoH volume; (C) the recovery of PSA and C18; (D) Effect of quantity of C18; Figure S2. Extraction ion chromatograms of melatonin; Figure S3. The expressional levels of potential candidate genes associated with melatonin content in sesame seed at different stages of development. Heat maps were constructed based on the log10-transformed RPKM values for each gene. The color scale for expression values is shown. DAF: days after flowering; Figure S4. Expression of SiWRKY67 in SiWRKY67-overexpressing transgenic hairy root lines. Error bars indicate SE based on three replicates. VC: empty vector control; Table S1. The list of 450 accessions sampled in the collection.
