# Supplementary material for: Variation in Melatonin Contents and Genetic Dissection of Melatonin Biosynthesis in Sesame
Source: Plants (Basel). 2022 Jul 31;11(15):2005. doi: 10.3390/plants11152005 (PMC9370803; doi:10.3390/plants11152005)
Supplement: Supplementary file 1 [file plants-11-02005-s001.zip › plants-1769365-supplementary.pdf]

Supplementary Materials

Figure S1. Optimization of sample pretreatment conditions (A) the extraction efficiency of Methanol (MeoH) and Acetonitrile (ACN)); (B) Effect of extraction solvent MeoH volume; (C) the recovery of PSA and C18; (D) Effect of quantity of C_{18}

Figure S2. Extraction ion chromatograms of melatonin

Figure S3. The expressional levels of potential candidate genes associated with melatonin content in sesame seed at different stage of development. Heat maps were constructed based on the log10-transformed RPKM values for each gene. The color scale for expression values is shown. DAF: days after flowering

Figure S4. Expression of SiWRKY67 in SiWRKY67-overexpressing transgenic hairy root lines. Error bars indicate SE based on three replicates. VC: empty vector control

Table S1. The list of 450 accessions sampled in the collection

Figure S1. Optimization of sample pretreatment conditions (A) the extraction efficiency of Methanol (MeoH) and Acetonitrile (ACN)); (B) Effect of extraction solvent MeoH volume; (C) the recovery of PSA and C_{18}; (D) Effect of quantity of C_{18}

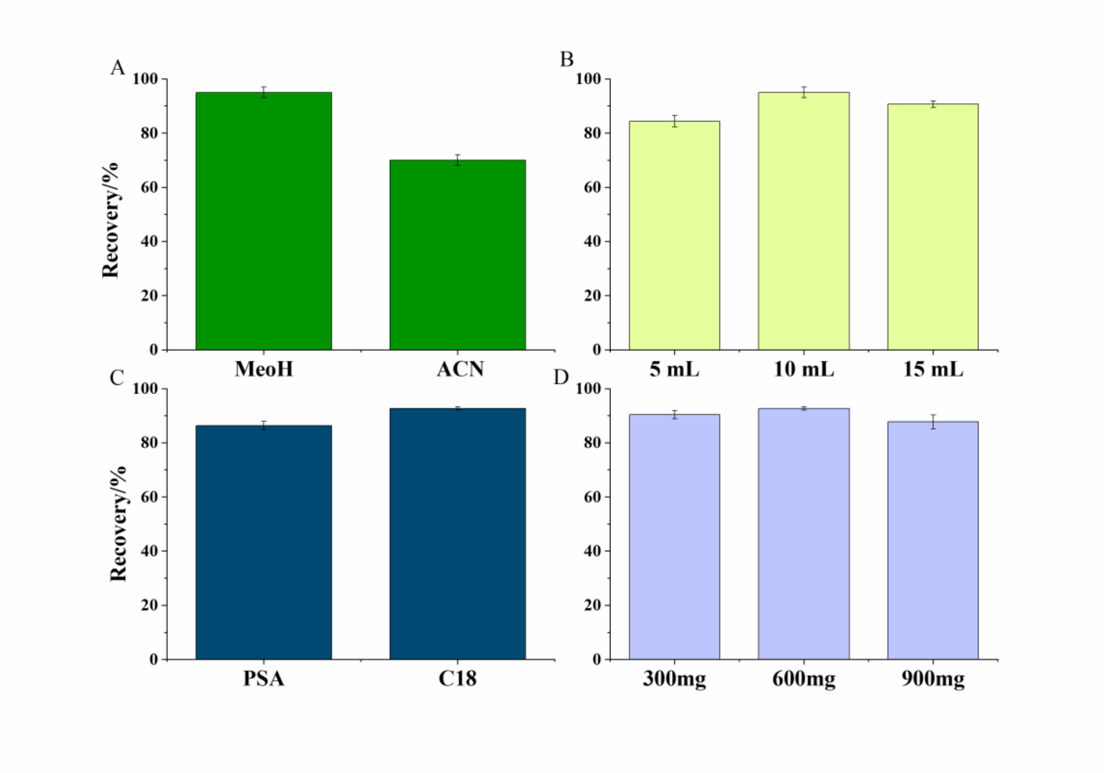

Figure S2. Extraction ion chromatograms of melatonin (A) and fragmentation pathways of melatonin (B)

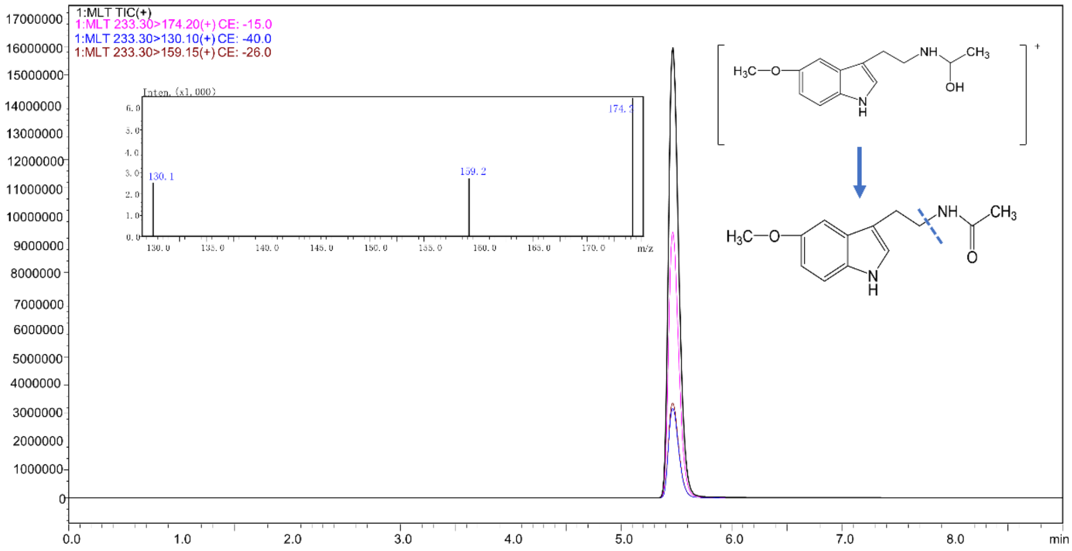

Figure S3. The expressional levels of potential candidate genes associated with melatonin content in sesame seed at different stage of development. Heat maps were constructed based on the log_{10}-transformed RPKM values for each gene. The color scale for expression values is shown. DAF: days after flowering.

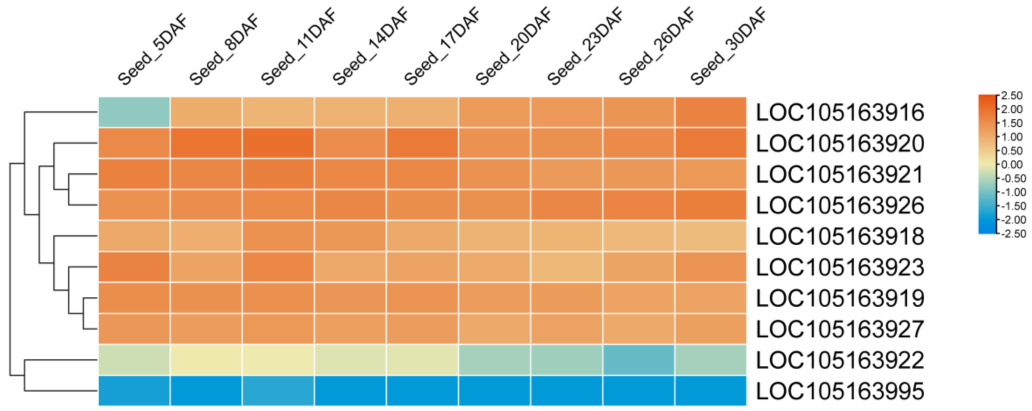

Figure S4. Expression of *SiWRKY67* in *SiWRKY67*-overexpressing transgenic hairy root lines. Error bars indicate SE based on three replicates. VC: empty vector control

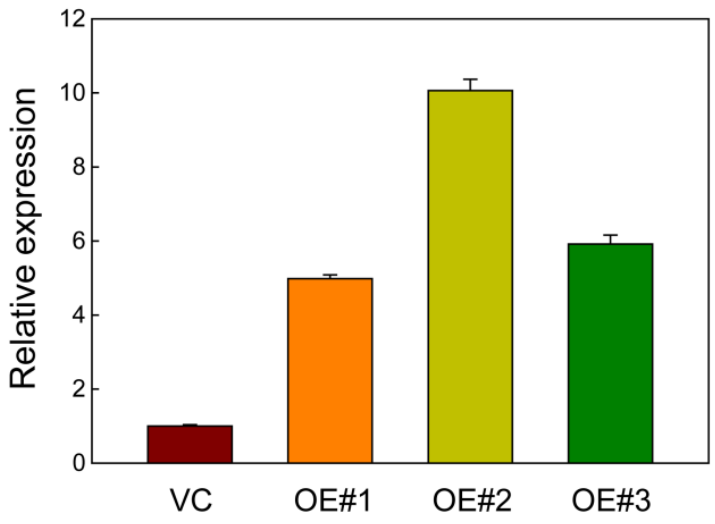

Table S1. The list of 450 accessions sampled in the collection

Accession ID	Accession name	Original producing area	Longitude(°)	Latitude(°)	Type	Grouping in neighbor-joining tree
G002	Zhima	China	116.33E	39.73N	Landrace	Southern-area group
G005	Zhima	China	116.58E	35.41N	Landrace	Northern-area group
G006	Xinbianwuhao	China	118.35E	35.19N	Landrace	Northern-area group
G007	Bagucha	China	115.09E	35.30N	Landrace	Northern-area group
G009	Heshangmao	China	116.30E	35.07N	Landrace	Northern-area group
G010	Baiyinzhima	China	121.38E	37.54N	Landrace	Northern-area group
G011	Zhuzhi1	China	NA	NA	Modern cultivar	Southern-area group
G013	Huangzhima	China	113.82E	34.03N	Landrace	Northern-area group
G014	Badacha	China	113.06E	33.87N	Landrace	Northern-area group
G015	Heizhima	China	114.86E	31.63N	Landrace	Southern-area group
G016	Baizhima	China	114.86E	34.06N	Landrace	Northern-area group
G017	Baicao	China	114.90E	33.44N	Landrace	Northern-area group
G019	Xiaozihuang	China	113.99E	33.15N	Landrace	Southern-area group
G020	Waizuihong	China	114.73E	32.34N	Landrace	Northern-area group
G024	Baizhima	China	118.58E	30.08N	Landrace	Southern-area group
G025	Zhima	China	118.43E	32.45N	Landrace	Northern-area group
G026	Guanyinma	China	114.11E	31.56N	Landrace	Northern-area group
G027	Baizhima	China	112.14E	32.05N	Landrace	Southern-area group
G028	Duijiaozhima	China	114.32E	30.35N	Landrace	Southern-area group
G029	786	China	NA	NA	Modern cultivar	Southern-area group
G030	Wujiaozhan	China	113.37E	31.72N	Landrace	Southern-area group
G032	Zhongzhi5hao	China	114.33E	30.35N	Landrace	Southern-area group
G033	Zhima	China	114.11E	31.56N	Landrace	Southern-area group

G034	Hongyangzhima	China	112.14E	32.05N	Landrace	Northern-area group
G035	Dikehuang	China	112.76E	32.13N	Landrace	Southern-area group
G036	Tanma	China	113.69E	31.26N	Landrace	Southern-area group
G043	Zhima	China	121.43E	29.28N	Landrace	Northern-area group
G045	Zhima	China	83.62E	46.52N	Landrace	Northern-area group
G047	Zhima	China	89.18E	42.94N	Landrace	Northern-area group
G048	Zhima	China	89.18E	42.94N	Landrace	Northern-area group
G049	Zhima	China	89.18E	42.94N	Landrace	Northern-area group
G051	Balengzhima	China	111.75E	37.27N	Landrace	Northern-area group
G053	Zhima	China	112.02E	37.44N	Landrace	Northern-area group
G054	Zhima	China	112.84E	35.51N	Landrace	Northern-area group
G055	Hezhima	China	111.08E	39.01N	Landrace	Southern-area group
G056	Baizhima	China	109.94E	34.80N	Landrace	Southern-area group
G057	Baizhima	China	110.15E	35.24N	Landrace	Southern-area group
G058	Zhima	China	108.24E	34.53N	Landrace	Southern-area group
G059	Zhima	China	108.25E	33.04N	Landrace	Northern-area group
G060	Yeyesan	China	109.03E	32.69N	Landrace	Northern-area group
G061	Zhima	China	109.43E	35.76N	Landrace	Southern-area group
G062	Zhima	China	110.87E	33.53N	Landrace	Southern-area group
G063	Xizhima	China	108.24E	34.53N	Landrace	Northern-area group
G064	Zhima	China	108.32E	33.32N	Landrace	Northern-area group
G065	Yizhima	China	109.59E	35.18N	Landrace	Northern-area group
G066	Baizhima	China	107.38E	34.35N	Landrace	Southern-area group
G067	Wucuolian	China	108.48E	34.30N	Landrace	Southern-area group
G069	Heizhima	China	117.93E	35.71N	Landrace	Northern-area group
G070	Henanyihao	China	113.66E	34.77N	Landrace	Southern-area group

G071	Huangzhima	China	118.41E	30.69N	Landrace	Southern-area group
G072	Zhima	China	115.86E	31.69N	Landrace	Southern-area group
G076	Hezhima	China	109.89E	31.08N	Landrace	Southern-area group
G077	Heizhima	China	105.38E	31.23N	Landrace	Southern-area group
G078	Zhuanzhulian-1	China	113.37E	31.72N	Landrace	Southern-area group
G079	Zhongzhi8	China	NA	NA	Modern cultivar	Southern-area group
G080	Sifangzhima	China	113.81E	29.26N	Landrace	Southern-area group
G082	Huoma	China	113.82E	31.62N	Landrace	Northern-area group
G083	Jinkouheizhima	China	120.75E	30.77N	Landrace	Southern-area group
G085	Mishuozhima	China	120.23E	29.26N	Landrace	Southern-area group
G087	Yingsangu	China	120.39E	27.51N	Landrace	Southern-area group
G088	Baizhima	China	120.96E	28.13N	Landrace	Southern-area group
G089	Zhima	China	117.58E	28.95N	Landrace	Southern-area group
G091	Zhima	China	115.89E	29.61N	Landrace	Southern-area group
G092	Zhima	China	NA	NA	Landrace	Southern-area group
G098	Baizhima	China	116.77E	23.46N	Landrace	Southern-area group
G102	Baizhima	China	114.27E	23.17N	Landrace	Southern-area group
G104	Zhima	China	123.34E	42.75N	Landrace	Northern-area group
G107	Zhima	China	122.45E	42.31N	Landrace	Northern-area group
G110	Bawangbian	China	119.90E	42.28N	Landrace	Northern-area group
G111	Jiaopanzhima	China	111.78E	37.14N	Landrace	Northern-area group
G116	Baizhima	China	109.15E	33.43N	Landrace	Northern-area group
G117	Hongzhima	China	109.94E	34.80N	Landrace	Southern-area group
G118	Huangzhima	China	110.87E	33.53N	Landrace	Northern-area group
G119	Heizhima	China	119.17E	34.61N	Landrace	Southern-area group
G120	Huangzhima	China	116.27E	30.42N	Landrace	Southern-area group

G121	Zhima	China	116.11E	30.15N	Landrace	Southern-area group
G122	Muzhenbai	China	117.86E	30.64N	Landrace	Northern-area group
G123	Zhufangzhima	China	115.86E	31.69N	Landrace	Southern-area group
G125	Zhangdianbai	China	116.49E	31.75N	Landrace	Southern-area group
G126	Dushanhei	China	119.42E	30.89N	Landrace	Southern-area group
G127	Zhima	China	116.97E	33.64N	Landrace	Southern-area group
G129	Heizhima	China	109.62E	31.41N	Landrace	Southern-area group
G131	Wuyahei	China	109.62E	31.41N	Landrace	Southern-area group
G132	Duozhima	China	108.89E	30.97N	Landrace	Southern-area group
G134	Zhima	China	109.71E	32.33N	Landrace	Southern-area group
G135	Mazhima	China	110.76E	31.24N	Landrace	Southern-area group
G136	Mazhima	China	110.73E	32.06N	Landrace	Northern-area group
G137	Hezhima	China	110.76E	31.24N	Landrace	Southern-area group
G139	Liujiaobaizhima	China	121.37E	31.11N	Landrace	Southern-area group
G142	Zichangma	China	117.90E	28.43N	Landrace	Southern-area group
G143	Haihuiheizhima	China	115.89E	29.61N	Landrace	Southern-area group
G144	Zihuahei	China	115.10E	29.26N	Landrace	Southern-area group
G146	Wenzhubaizhima	China	114.78E	26.46N	Landrace	Southern-area group
G147	Zhima	China	109.85E	29.01N	Landrace	Southern-area group
G148	Zhima	China	106.82E	27.54N	Landrace	Southern-area group
G150	Baizhima	China	119.30E	26.08N	Landrace	Southern-area group
G151	Zhima	China	NA	NA	Landrace	Southern-area group
G152	Zhima	China	NA	NA	Landrace	Southern-area group
G153	Bachazhima	China	101.99E	24.08N	Landrace	Southern-area group
G154	Baizhima	China	105.33E	24.78N	Landrace	Southern-area group
G160	Baizhima	China	110.19E	20.1N	Landrace	Southern-area group

G163	Tainanheixuan1hao	China	120.31E	23.31N	Landrace	Southern-area group
G164	Heizhima	China	114.88E	33.73N	Landrace	Southern-area group
G165	Sangenqiang	China	114.88E	33.73N	Landrace	Northern-area group
G166	Yibabian	China	113.66E	35.27N	Landrace	Northern-area group
G167	Zhima	China	113.81E	36.07N	Landrace	Southern-area group
G168	Youhulu	China	113.32E	32.72N	Landrace	Northern-area group
G170	Waizuibai	China	114.77E	34.55N	Landrace	Northern-area group
G171	Baizhima	China	114.26E	33.27N	Landrace	Northern-area group
G172	Liutiaoqing	China	114.52E	33.79N	Landrace	Northern-area group
G173	Erlanghuazhima	China	114.02E	33.39N	Landrace	Northern-area group
G174	Baizhima	China	112.36E	32.52N	Landrace	Northern-area group
G175	Yiyesan	China	112.36E	32.52N	Landrace	Northern-area group
G177	Baizhima	China	106.93E	33.00N	Landrace	Northern-area group
G178	Bantangzhima	China	117.86E	31.60N	Landrace	Southern-area group
G179	Baizhima	China	118.48E	31.56N	Landrace	Southern-area group
G180	Heizhima	China	117.47E	31.89N	Landrace	Southern-area group
G181	Youzhima	China	117.16E	31.72N	Landrace	Southern-area group
G182	Jiugugangcha	China	117.55E	32.86N	Landrace	Northern-area group
G183	Bawangbian	China	116.26E	32.34N	Landrace	Southern-area group
G184	Huangzhima	China	118.33E	30.92N	Landrace	Southern-area group
G185	Ningguohuangzhima	China	118.98E	30.62N	Landrace	Southern-area group
G187	Zhima	China	118.26E	32.10N	Landrace	Northern-area group
G188	Dayanhei	China	117.47E	30.21N	Landrace	Southern-area group
G190	Huangchizhima	China	118.57E	31.15N	Landrace	Southern-area group
G191	Youzhima	China	118.43E	29.87N	Landrace	Southern-area group
G192	Yingshangzhimaliuhao	China	116.25E	32.64N	Landrace	Southern-area group

G194	Tieqingzhima	China	110.11E	32.81N	Landrace	Northern-area group
G195	Huazhima	China	110.33E	33.69N	Landrace	Southern-area group
G196	Zhima	China	109.08E	34.53N	Landrace	Northern-area group
G197	Zhima	China	109.29E	37.96N	Landrace	Northern-area group
G198	Zhima	China	114.52E	33.79N	Landrace	Northern-area group
G199	Zhima	China	110.50E	38.02N	Landrace	Northern-area group
G201	Zhima	China	106.85E	34.89N	Landrace	Southern-area group
G202	Hongzhima	China	106.16E	33.33N	Landrace	Southern-area group
G204	Baizhima	China	114.02E	33.39N	Landrace	Northern-area group
G205	Heizhima	China	110.01E	36.58N	Landrace	Northern-area group
G206	Heizhima	China	108.97E	34.91N	Landrace	Southern-area group
G207	Zhima	China	109.52E	31.05N	Landrace	Southern-area group
G208	Baizhima	China	108.42E	31.19N	Landrace	Southern-area group
G209	Xiaoheizi	China	108.35E	30.84N	Landrace	Southern-area group
G210	Zhuanjiaolou	China	105.38E	31.23N	Landrace	Southern-area group
G211	Baihuama	China	108.89E	30.97N	Landrace	Southern-area group
G212	Zhima	China	110.40E	31.03N	Landrace	Northern-area group
G213	Xiniujiao	China	111.65E	32.27N	Landrace	Southern-area group
G214	Yangzhima	China	111.68E	32.39N	Landrace	Southern-area group
G215	Zhuganqing	China	114.37E	30.88N	Landrace	Northern-area group
G216	Yesanjiao	China	113.90E	29.98N	Landrace	Southern-area group
G217	Zhima	China	112.19E	30.35N	Landrace	Northern-area group
G218	Silunzi	China	112.20E	31.03N	Landrace	Southern-area group
G219	Qiezhima	China	115.39E	30.79N	Landrace	Southern-area group
G221	Zhima	China	112.20E	31.03N	Landrace	Southern-area group
G222	Fazhima	China	113.16E	30.66N	Landrace	Southern-area group

G223	Baizhima	China	110.68E	30.20N	Landrace	Southern-area group
G224	Fupima	China	113.91E	30.93N	Landrace	Southern-area group
G228	Huangzhima11hao	China	112.59E	31.17N	Landrace	Southern-area group
G229	Baizhima	China	110.69E	31.00N	Landrace	Southern-area group
G231	Zhima	China	113.11E	29.14N	Landrace	Southern-area group
G232	Baizhima	China	116.66E	28.99N	Landrace	Southern-area group
G233	Qiancengta	China	116.66E	28.99N	Landrace	Southern-area group
G236	Duolengzhima	China	117.20E	28.29N	Landrace	Southern-area group
G238	Duoshuoguozhima	China	115.89E	29.61N	Landrace	Southern-area group
G239	Dayanghuangzhima	China	116.30E	27.93N	Landrace	Northern-area group
G243	Heizhima	China	117.90E	28.43N	Landrace	Southern-area group
G244	Qiuheimaxuan3	China	117.90E	28.43N	Landrace	Southern-area group
G246	Heizhima	China	115.10E	29.26N	Landrace	Southern-area group
G250	Zhima	China	116.86E	38.31N	Landrace	Northern-area group
G251	Xinshengyihao	China	116.65E	36.95N	Landrace	Northern-area group
G252	Dabacha	China	119.40E	35.99N	Landrace	Southern-area group
G254	Daqingjie	China	118.54E	36.51N	Landrace	Northern-area group
G255	Yitiaobian	China	118.34E	35.07N	Landrace	Northern-area group
G256	Zhima	China	117.52E	36.71N	Landrace	Northern-area group
G258	Lvjingzhima	China	123.18E	47.34N	Landrace	Northern-area group
G262	Baizhima	China	82.99E	46.75N	Landrace	Northern-area group
G263	Batongzhima	China	116.09E	38.71N	Landrace	Northern-area group
G265	Baizhima	China	112.92E	35.79N	Landrace	Northern-area group
G266	Fenchazhima	China	111.22E	35.14N	Landrace	Northern-area group
G267	Zhima	China	113.04E	36.53N	Landrace	Northern-area group
G268	Fenchazhima	China	111.47E	35.64N	Landrace	Northern-area group

G269	Heizhima	China	112.34E	37.61N	Landrace	Northern-area group
G270	Datoubang	China	121.41E	29.65N	Landrace	Southern-area group
G271	Liutiaoqing	China	120.43E	29.05N	Landrace	Southern-area group
G274	Baizhima	China	108.74E	27.22N	Landrace	Southern-area group
G275	Baizhima	China	105.18E	25.44N	Landrace	Southern-area group
G276	Baizhima	China	108.49E	22.75N	Landrace	Southern-area group
G277	Baizhima	China	109.23E	23.73N	Landrace	Southern-area group
G278	Shangningaizhima	China	108.66E	24.06N	Landrace	Southern-area group
G283	Liusuozhima	China	119.49E	28.45N	Landrace	Northern-area group
G286	Zhima	China	106.82E	28.14N	Landrace	Southern-area group
G287	Baizhima	China	108.84E	27.69N	Landrace	Southern-area group
G288	Heizhima	China	107.88E	25.41N	Landrace	Southern-area group
G289	Baizhima	China	117.61E	24.12N	Landrace	Southern-area group
G292	Baizhima	China	107.98E	22.15N	Landrace	Southern-area group
G293	Heizhima	China	111.27E	24.82N	Landrace	Southern-area group
G297	Zhima	China	121.79E	41.60N	Landrace	Northern-area group
G298	Zhima	China	120.33E	40.33N	Landrace	Northern-area group
G300	Bawangbian	China	117.06E	39.38N	Landrace	Northern-area group
G301	Dengmingsizhima	China	116.53E	37.88N	Landrace	Northern-area group
G302	Zihuazhima	China	114.83E	38.04N	Landrace	Northern-area group
G303	Sangucha	China	114.95E	36.78N	Landrace	Northern-area group
G304	Xiaobacha	China	115.97E	37.34N	Landrace	Northern-area group
G305	Zhima	China	115.02E	37.21N	Landrace	Northern-area group
G306	Wenrenheizhima	China	115.48E	38.76N	Landrace	Northern-area group
G307	Laohongzhima	China	111.48E	33.14N	Landrace	Northern-area group
G308	Baizhima	China	112.58E	35.09N	Landrace	Northern-area group

G309	Yeersan	China	114.63E	32.96N	Landrace	Northern-area group
G323	Zhima	China	90.21E	42.86N	Landrace	Northern-area group
G325	Zhima	China	86.56E	42.06N	Landrace	Northern-area group
G327	Zhima	China	75.86E	39.38N	Landrace	Northern-area group
G328	Longshanzhima	China	118.77E	32.05N	Landrace	Southern-area group
G329	Chama	China	117.19E	34.27N	Landrace	Northern-area group
G330	Deleilei	China	119.42E	32.39N	Landrace	Northern-area group
G332	Zhima	China	95.26E	25.22N	Landrace	Southern-area group
G333	Aijiaojinhuangma	China	116.26E	28.36N	Landrace	Southern-area group
G334	Baizhima	China	105.22E	25.83N	Landrace	Northern-area group
G336	Luorongchuanma	China	109.74E	24.49N	Landrace	Southern-area group
G337	Chengxiangheizhima	China	107.91E	22.64N	Landrace	Southern-area group
G338	Heizhima	China	110.25E	21.38N	Landrace	Southern-area group
G339	Silengzhima	China	115.89E	24.57N	Landrace	Southern-area group
G340	Baizhima	China	110.32E	19.70N	Landrace	Southern-area group
G341	Hanguangchanshensileng	China	113.40E	24.17N	Landrace	Southern-area group
G345	Xinyangbaizhima	China	120.26E	33.77N	Landrace	Northern-area group
G346	Baizhima	China	120.14E	33.39N	Landrace	Southern-area group
G347	Baizhima	China	119.57E	31.75N	Landrace	Southern-area group
G348	Heizhima	China	120.46E	33.20N	Landrace	Southern-area group
G349	Chama	China	121.08E	32.09N	Landrace	Southern-area group
G350	Luanshanheizhima	China	113.34E	27.00N	Landrace	Southern-area group
G351	Heizhima	China	113.57E	28.71N	Landrace	Southern-area group
G352	Shuanghuzhima	China	111.12E	29.43N	Landrace	Southern-area group
G353	Zhima	China	111.48E	28.90N	Landrace	Southern-area group
G354	Tianwanhongzhima	China	110.18E	28.02N	Landrace	Southern-area group

G355	Zhima	China	110.84E	26.43N	Landrace	Southern-area group
G356	Hukouzhima	China	113.55E	26.80N	Landrace	Southern-area group
G357	Zhima	China	110.40E	28.46N	Landrace	Southern-area group
G359	Heizhima	China	118.14E	24.74N	Landrace	Southern-area group
G360	Heizhima	China	117.60E	26.22N	Landrace	Southern-area group
G361	Baizhima	China	119.99E	26.88N	Landrace	Northern-area group
G362	Baizhima	China	118.03E	27.76N	Landrace	Northern-area group
G364	Heizhima	China	117.33E	27.54N	Landrace	Southern-area group
G366	Zhima	China	101.54E	25.03N	Landrace	Southern-area group
G368	Zhima	China	100.56E	25.48N	Landrace	Southern-area group
G370	Baizhima	China	101.67E	26.06N	Landrace	Southern-area group
G371	Zhima(248hao)	China	102.74E	23.17N	Landrace	Southern-area group
G372	Zhima	China	101.28E	25.20N	Landrace	Southern-area group
G377	Baizhima	China	107.25E	31.93N	Landrace	Southern-area group
G378	Mazhima	China	107.72E	31.36N	Landrace	Southern-area group
G379	Zhima	China	106.29E	32.22N	Landrace	Southern-area group
G380	Zhuanjiaolou	China	106.83E	32.36N	Landrace	Southern-area group
G381	Zhima	China	103.57E	28.26N	Landrace	Southern-area group
G382	Huangzhima	China	105.23E	32.58N	Landrace	Southern-area group
G383	Zhima	China	104.44E	31.83N	Landrace	Southern-area group
G384	Zhima	China	102.17E	28.55N	Landrace	Southern-area group
G385	Zhima	China	101.51E	27.42N	Landrace	Southern-area group
G386	Zhima	China	104.53E	32.41N	Landrace	Southern-area group
G387	Zhima	China	103.13E	28.33N	Landrace	Southern-area group
G388	Zhima	China	103.57E	28.26N	Landrace	Southern-area group
G389	Bendizhima	China	101.55E	26.91N	Landrace	Southern-area group

G390	Heizhima	China	105.83E	32.44N	Landrace	Southern-area group
G391	Heizhima	China	102.11E	26.89N	Landrace	Southern-area group
G392	99-2188	China	NA	NA	Modern cultivar	Southern-area group
G393	Zhima	China	122.07E	46.06N	Landrace	Southern-area group
G396	Zhima	China	121.55E	45.38N	Landrace	Northern-area group
G397	Ezhi1hao	China	NA	NA	Modern cultivar	Southern-area group
G398	Ezhi2hao	China	NA	NA	Modern cultivar	Southern-area group
G399	Ezhi5hao	China	NA	NA	Modern cultivar	Southern-area group
G400	Ezhi6hao	China	NA	NA	Modern cultivar	Southern-area group
G401	Zhongfengzhiyihao	China	NA	NA	Modern cultivar	Southern-area group
G402	Zhongzhi10	China	NA	NA	Modern cultivar	Southern-area group
G403	Zhongzhi11	China	NA	NA	Modern cultivar	Southern-area group
G404	Zhongzhi16	China	NA	NA	Modern cultivar	Northern-area group
G405	98N09	China	NA	NA	Modern cultivar	Southern-area group
G406	Shanzhi3hao	China	NA	NA	Modern cultivar	Southern-area group
G408	Jinhuangma	China	116.26E	28.36N	Landrace	Southern-area group
G409	UCLA83	Venezuela	69.52W	9.52N	NA	Southern-area group
G410	VIR 80	Uzbekistan	69.13E	41.16N	NA	Northern-area group
G411	VIR 136	Armenia	44.51E	40.11N	NA	Northern-area group
G412	VIR 145	Armenia	44.51E	40.11N	NA	Northern-area group
G413	VIR 146	America	97.44W	30.17N	NA	Northern-area group
G414	VIR 202	Turkey	30.05E	37.12N	NA	Southern-area group
G415	VIR 207	Turkey	30.05E	37.12N	NA	Northern-area group
G417	VIR 231	Greece	23.25E	37.05N	NA	Southern-area group
G418	VIR 825	Uzbekistan	69.13E	41.16N	NA	Northern-area group
G419	VIR 896	Uzbekistan	69.13E	41.16N	NA	Northern-area group

G420	VIR 899	Russia	39.26E	45.22N	NA	Southern-area group
G421	VIR 908	Turkmenistan	58.38E	37.95N	NA	Northern-area group
G424	VIR 1767	Russia	39.26E	45.22N	NA	Northern-area group
G427	ZM11-18	Burma	96.09E	16.48N	NA	Southern-area group
G428	Jinkouzhima	Tanzania	37.51E	7.13S	NA	Southern-area group
G429	Tashigan122	Uzbekistan	69.13E	41.16N	NA	Southern-area group
G432	Heizhima	Vietnam	103.53E	21.57N	NA	Southern-area group
G436	K1	Guinea	13.40W	9.32N	NA	Southern-area group
G437	Heizhima	Mexico	102.33W	23.38N	NA	Northern-area group
G438	Guba-3	Cuba	82.23W	23.70N	NA	Southern-area group
G439	341(34)	Japan	136.50E	35.12N	NA	Northern-area group
G440	10(27)	Japan	136.50E	35.12N	NA	Northern-area group
G443	523(41)	Japan	136.50E	35.12N	NA	Northern-area group
G444	Teras	Mexico	99.55W	25.25N	NA	Southern-area group
G445	TKV-334(22)	Japan	136.50E	35.12N	NA	Northern-area group
G446	TKV-309(23)	Japan	136.50E	35.12N	NA	Northern-area group
G448	723(38)	Japan	136.50E	35.12N	NA	Northern-area group
G449	U.C.R/82NO209shat	America	97.32W	35.29N	NA	Southern-area group
G450	Margo short	Mozambique	32.40E	25.11S	NA	Northern-area group
G452	Oro short	Mozambique	32.40E	25.11S	NA	Southern-area group
G454	384(7)	Japan	136.50E	35.12N	NA	Northern-area group
G456	342(5)	Japan	136.50E	35.12N	NA	Northern-area group
G458	725(39)	Japan	136.50E	35.12N	NA	Northern-area group
G459	301(3)	Japan	136.50E	35.12N	NA	Northern-area group
G460	Oro tall	Mozambique	32.40E	25.11S	NA	Southern-area group
G461	Zirra	Mozambique	32.35E	25.58S	NA	Northern-area group

G465	Teras	Mexico	99.55W	25.25N	NA	Northern-area group
G466	Suke No.5	Mozambique	32.35E	25.58S	NA	Northern-area group
G468	Inamar	Mozambique	32.35E	25.58S	NA	Southern-area group
G469	Oro 9/71	Mozambique	32.35E	25.58S	NA	Southern-area group
G475	U.C.R/82NO16NS	America	86.37W	34.42N	NA	Southern-area group
G477	U.C.R/82NO8NS	America	97.44W	30.17N	NA	Southern-area group
G479	Nian(Pungnyon)	South Korea	125.48E	35.38N	NA	Southern-area group
G481	Danbai	South Korea	126.58E	37.33N	NA	Southern-area group
G483	Guangchan	South Korea	126.58E	37.33N	NA	Southern-area group
G484	Anchan	South Korea	126.58E	37.33N	NA	Northern-area group
G486	Rucheng	South Korea	127.34E	36.05N	NA	Northern-area group
G488	Jinback	South Korea	127.07E	34.58N	NA	Northern-area group
G489	Yeoju-1	South Korea	127.32E	37.14N	NA	Southern-area group
G494	NW1	Thailand	99.29E	16.12N	NA	Southern-area group
G495	His-173	Philippines	120.35E	17.20N	NA	Southern-area group
G500	GMS-1	America	112.05W	33.31N	NA	Southern-area group
G501	Potepye	Burma	96.06E	19.45N	NA	Southern-area group
G502	Patiae-1	Burma	96.50E	21.58N	NA	Southern-area group
G504	91-2191	Israel	34.46E	32.04N	NA	Southern-area group
G505	No.835-1	Bangladesh	90.24E	23.42N	NA	Southern-area group
G507	91-2299	Israel	34.46E	32.04N	NA	Southern-area group
G510	393-3Bo	Thailand	101.01E	15.53N	NA	Southern-area group
G512	Magwe 719	Burma	96.50E	21.58N	NA	Southern-area group
G514	Maeshae	Burma	95.39E	19.08N	NA	Southern-area group
G516	Ses-11	Bangladesh	90.24E	23.42N	NA	Southern-area group
G517	No.016211	Italy	12.29E	41.53N	NA	Northern-area group

G519	METHILA	India	79.19E	28.10N	NA	Southern-area group
G520	HNNAN-NI	India	77.22E	24.34N	NA	Southern-area group
G522	SINYA DANA-3	India	75.58E	21.06N	NA	Southern-area group
G524	Boder Racet	Thailand	100.31E	13.45N	NA	Southern-area group
G527	EC－343404	India	77.32E	10.27N	NA	Northern-area group
G528	EC－342782	India	79.07E	14.11N	NA	Northern-area group
G531	EC－355640	India	87.31E	22.55N	NA	Southern-area group
G532	EC－355666	India	84.02E	20.46N	NA	Southern-area group
G534	EC－350643	India	76.20E	19.12N	NA	Northern-area group
G535	EC－357032	India	76.13E	12.46N	NA	Northern-area group
G538	S.J	Mexico	108.30W	29.34N	NA	Southern-area group
G539	68·544	Nicaragua	86.15W	12.08N	NA	Southern-area group
G540	726	Afghanistan	69.10E	34.31N	NA	Southern-area group
G542	68-546	Nicaragua	86.15W	12.08N	NA	Southern-area group
G551	1034	Israel	34.46E	32.04N	NA	Southern-area group
G556	Zhima9	Vietnam	105.51E	21.02N	NA	Southern-area group
G557	Zhima1	Vietnam	105.11E	20.25N	NA	Southern-area group
G558	Zhima18	Vietnam	106.41E	17.12N	NA	Southern-area group
G559	Zhima21	Vietnam	105.51E	21.02N	NA	Southern-area group
G561	Me den	America	112.05W	33.31N	NA	Southern-area group
G565	Luozhi21	China	NA	NA	Modern cultivar	Southern-area group
G574	Zhima101	Vietnam	105.51E	21.02N	NA	Southern-area group
G577	Zhima10	Vietnam	106.11E	20.32N	NA	Southern-area group
G580	Zhima40	Vietnam	106.51E	21.27N	NA	Northern-area group
G583	Ezhi7hao	China	NA	NA	Modern cultivar	Southern-area group
G590	NA	United Arab Emirates	54.22E	24.28N	NA	Southern-area group

G591	K1	Guinea	13.40W	9.32N	NA	Southern-area group
G592	K1	Guinea	13.40W	9.32N	NA	Southern-area group
G593	K2	Guinea	13.40W	9.32N	NA	Southern-area group
G594	K2	Guinea	13.40W	9.32N	NA	Southern-area group
G596	Calinda	America	112.05W	33.31N	NA	Northern-area group
G598	Guba-1	Cuba	82.23W	23.07N	NA	Southern-area group
G599	Guba-2	Cuba	82.23W	23.07N	NA	Southern-area group
G600	Guba-3	Cuba	82.23W	23.07N	NA	Southern-area group
G601	Guba-4	Cuba	82.23W	23.07N	NA	Southern-area group
G602	VIR 741	Uzbekistan	69.13E	41.16N	NA	Northern-area group
G603	Zhima(8131)	China	117.90E	28.43N	Landrace	Southern-area group
G604	Zhongzhi15hao	China	NA	NA	Modern cultivar	Southern-area group
G605	Yiyangbai	China	112.17E	34.51N	Landrace	Southern-area group
G606	Zihuayeersan	China	114.26E	33.27N	Landrace	Northern-area group
G611	H98	China	115.93E	30.08N	Landrace	Southern-area group
G613	Zhima	China	116.34E	35.41N	Landrace	Northern-area group
G616	Jizhi1hao	China	NA	NA	Modern cultivar	Southern-area group
G617	Zhima	Vietnam	106.11E	17.47N	NA	Northern-area group
G619	Shuiyuan117	South Korea	127.14E	37.04N	NA	Southern-area group
G620	Xiangheizhi2078	China	NA	NA	Modern cultivar	Southern-area group
G621	Silengcao	China	115.81E	32.90N	Landrace	Southern-area group
G622	Jinzhi2hao	China	NA	NA	Modern cultivar	Northern-area group
G623	725	Afghanistan	69.10E	34.31N	NA	Southern-area group
G629	K1	Guinea	13.40W	9.32N	NA	Southern-area group
G630	Suke No.5	Mozambique	32.35E	25.58S	NA	Southern-area group
G632	Baizhima	China	110.23E	32.23N	Landrace	Southern-area group

G633	Zhongzhi12	China	NA	NA	Modern cultivar	Southern-area group
G634	Zhongzhi1hao	China	NA	NA	Modern cultivar	Southern-area group
G636	Yuzhi4hao	China	NA	NA	Modern cultivar	Southern-area group
G637	Yuzhi8hao	China	NA	NA	Modern cultivar	Southern-area group
G638	Zhengzhi13	China	NA	NA	Modern cultivar	Southern-area group
G639	Zhengzhi14	China	NA	NA	Modern cultivar	Southern-area group
G640	Luozhi12	China	NA	NA	Modern cultivar	Southern-area group
G641	Luozhi16	China	NA	NA	Modern cultivar	Southern-area group
G643	Zhuzhi11	China	NA	NA	Modern cultivar	Southern-area group
G644	Zhuzhi18	China	NA	NA	Modern cultivar	Southern-area group
G646	Suxianzhima	China	116.58E	33.37N	Landrace	Northern-area group
G647	Wanzhi1hao	China	NA	NA	Modern cultivar	Southern-area group
G648	Wanzhi2hao	China	NA	NA	Modern cultivar	Southern-area group
G649	Wucuolian	China	108.48E	34.30N	Landrace	Southern-area group
G650	Jizhi2hao	China	NA	NA	Modern cultivar	Northern-area group
G651	Jizhi1hao	China	NA	NA	Modern cultivar	Southern-area group
G652	Jizhi2hao	China	NA	NA	Modern cultivar	Southern-area group
G653	Liaopinzhi1	China	NA	NA	Modern cultivar	Northern-area group
G654	Liaopinzhi2	China	NA	NA	Modern cultivar	Northern-area group
G656	Ezhi4hao	China	NA	NA	Modern cultivar	Southern-area group
G657	Hangzhi2hao	China	NA	NA	Modern cultivar	Southern-area group
G658	Zhongzhi14	China	NA	NA	Modern cultivar	Southern-area group
G659	Zhongzhi17	China	NA	NA	Modern cultivar	Southern-area group
G660	Zhongzhi28	China	NA	NA	Modern cultivar	Southern-area group
G661	Zhongzhi3hao	China	NA	NA	Modern cultivar	Southern-area group
G662	Zhongzhi5hao	China	114.33E	30.35N	Landrace	Southern-area group

G663	Zhongzhi7hao	China	NA	NA	Modern cultivar	Southern-area group
G664	Zhongzhi8hao	China	114.33E	30.35N	Landrace	Southern-area group
G665	Yuzhi11hao	China	NA	NA	Modern cultivar	Southern-area group
G666	Yuzhi14	China	NA	NA	Modern cultivar	Southern-area group
G668	Xin5hao	China	NA	NA	Modern cultivar	Southern-area group
G669	Yuzhi10hao	China	NA	NA	Modern cultivar	Southern-area group
G670	Yuzhi5hao	China	NA	NA	Modern cultivar	Southern-area group
G671	Yuzhi6hao	China	NA	NA	Modern cultivar	Southern-area group
G672	Yuzhi7hao	China	NA	NA	Modern cultivar	Southern-area group
G673	Zhengzhi04C85	China	NA	NA	Modern cultivar	Southern-area group
G674	Zhengzhi12hao	China	NA	NA	Modern cultivar	Southern-area group
G675	Zhengzhi2010	China	NA	NA	Modern cultivar	Southern-area group
G678	Zhengzhi97C01	China	NA	NA	Modern cultivar	Southern-area group
G680	Luozhi15	China	NA	NA	Modern cultivar	Southern-area group
G681	Luozhi18hao	China	NA	NA	Modern cultivar	Southern-area group
G682	Luozhi19hao	China	NA	NA	Modern cultivar	Southern-area group
G683	Yuzhi15	China	NA	NA	Modern cultivar	Southern-area group
G684	Yuzhi2018	China	NA	NA	Modern cultivar	Southern-area group
G685	Zhuzhi14hao	China	NA	NA	Modern cultivar	Southern-area group
G686	Zhuzhi15hao	China	NA	NA	Modern cultivar	Southern-area group
G687	Zhuzhi16hao	China	NA	NA	Modern cultivar	Southern-area group
G688	Zhu0019	China	NA	NA	Modern cultivar	Southern-area group
G689	Wanzhi3hao	China	NA	NA	Modern cultivar	Southern-area group
G691	Xiongzhi1hao	China	NA	NA	Modern cultivar	Southern-area group
G692	Ji951	China	NA	NA	Modern cultivar	Northern-area group
G693	Jihangzhi1hao	China	NA	NA	Modern cultivar	Northern-area group

G695	Jizhi5hao	China	NA	NA	Modern cultivar	Northern-area group
G696	Jizhi6hao	China	NA	NA	Modern cultivar	Northern-area group
G697	Jingzhi1hao	China	NA	NA	Modern cultivar	Southern-area group
G698	Liaozhi2hao	China	NA	NA	Modern cultivar	Southern-area group
G699	Ningzhi1hao	China	NA	NA	Modern cultivar	Southern-area group
G700	Fenzhi2hao	China	NA	NA	Modern cultivar	Southern-area group
G701	Zhongzhi18	China	NA	NA	Modern cultivar	Southern-area group
G702	Zhongzhi19	China	NA	NA	Modern cultivar	Southern-area group
G703	Zhongzhi20	China	NA	NA	Modern cultivar	Southern-area group
G704	Zhuzhi20hao	China	NA	NA	Modern cultivar	Southern-area group
G705	Zhongzhi2771	China	NA	NA	Modern cultivar	Southern-area group
